# Short‐Term Unacylated Ghrelin Administration Improves Pyruvate Tolerance Without Chronically Altering Skeletal Muscle, Liver, or Adipose Tissue Insulin Response in Healthy Rats

**DOI:** 10.1096/fj.202604205R

**Published:** 2026-11-02

**Authors:** Nicole M. Notaro, Sofia F. Prizio, Brielle R. Caruso, David J. Dyck

**Affiliations:** ^1^ Department of Human Health Sciences University of Guelph Guelph Ontario Canada

**Keywords:** adipose tissue, gluconeogenic proteins, glucose uptake, insulin signaling, lipolytic proteins, liver, skeletal muscle, unacylated ghrelin

## Abstract

Unacylated ghrelin (unAG) is a metabolically active hormone, acutely stimulating fatty acid oxidation in skeletal muscle while promoting lipogenesis and inhibiting lipolysis in adipose tissue. Given these acute effects, we hypothesized that repeated unAG administration would improve skeletal muscle insulin response and that this would be associated with reduced muscle lipid storage. We treated healthy male Sprague–Dawley rats with twice‐daily subcutaneous unAG (200 μg; 400 μg total daily) for 5 days. UnAG treatment did not alter glucose uptake or AKT activation basally, or in response to low or high insulin concentrations when assessed ex vivo in oxidative or glycolytic skeletal muscle, or when muscle was pre‐exposed to a high‐palmitate stress. Protein content of mitochondrial markers (CPT1B, citrate synthase, COXIV) also remained unchanged with treatment in both muscle types. Interestingly, UnAG‐treated animals had a reduced glycemic response to pyruvate administration; however, content of hepatic glucose production‐associated proteins and liver AKT response to insulin remained unchanged. UnAG treatment tended to increase LPL protein content in visceral adipose tissue (*p* = 0.07), but did not alter AKT or HSL phosphorylation in response to an insulin bolus in either subcutaneous or visceral adipose tissue. Finally, triacylglycerol content in the red and white gastrocnemius muscle, as well as in the liver, was unchanged by unAG administration. Overall, these findings indicate that short‐term unAG treatment improves pyruvate tolerance, but that it does not exert persistent metabolic changes to skeletal muscle, liver, or adipose tissue substrate metabolism in healthy rats.

## Graphical abstract

Unacylated ghrelin (unAG) has been previously shown to acutely increase fatty acid oxidation (FAO) in skeletal muscle. 5 days of unAG administration to healthy male rats improved pyruvate tolerance, however this was not associated with changes in hepatic glucose‐production associated proteins. Treatment also did not have any effect on muscle or liver TAG content, skeletal muscle glucose uptake under basal, low‐, or high‐insulin stimulated conditions, or under acute high fat overload. It also did not alter insulin signaling in muscle, liver, or adipose tissue, or adipose tissue lipolytic signaling. There was a trend towards increased LPL content in VAT with unAG treatment. Overall, short‐term unAG administration improves pyruvate tolerance without causing any persistent changes in skeletal muscle, liver, or adipose substrate metabolism. COXIV, cytochrome c oxidase subunit IV; CPT1B, carnitine palmitoyltransferase 1B; CS, citrate synthase; EDL, extensor digitorum longus; G6Pase, glucose‐6‐phosphatase; HSL, hormone sensitive lipase; LPL, lipoprotein lipase; MCT1, monocarboxylate transporter 1; PEPCK, phosphoenolpyruvate carboxykinase; SAT, subcutaneous adipose tissue; TAG, triacylglycerol; VAT, visceral adipose tissue. Created with BioRender.com.

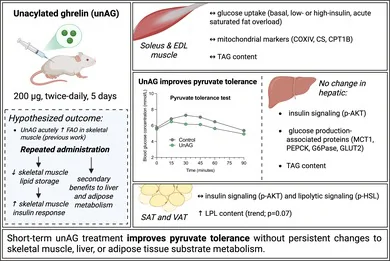

## 1 Introduction

Unacylated ghrelin (unAG) is a gut‐derived hormone released into the circulation prior to entrained mealtimes in tandem with its acylated counterpart (AG) [1, 2]. UnAG lacks the serine‐3 octanoyl modification required for interaction with the growth hormone secretagogue receptor 1a (GHSR1a) [3] and does not possess the canonical orexigenic effect attributed to AG [4]. Despite accounting for the majority of circulating ghrelin in healthy humans [5], unAG was traditionally considered the biologically inactive isoform. While a growing body of work has found unAG to alter glucose and lipid metabolism in several peripheral tissues [6, 7, 8, 9, 10], its physiological role remains to be fully understood.

Several studies have explored the effects of repeated AG administration on peripheral tissue metabolism [11, 12, 13, 14, 15], but relatively little is known about the impact of chronic unAG treatment. Available evidence suggests an anti‐diabetogenic effect of unAG, with repeated administration for 4 days resulting in increased insulin‐stimulated glucose uptake and AKT activation in rat skeletal muscle [8]. Additionally, unAG overexpression has been demonstrated to improve insulin response in normal [16] and high‐fat [8] fed mice. Despite this insulin‐sensitizing effect with chronic treatment, findings from our laboratory do not support a direct acute effect of unAG on skeletal muscle insulin response in the absence of fatty acids [17]. Importantly, however, the presence of unAG in a high saturated fatty acid buffer, which would normally result in acute impairments to the actions of insulin, has been demonstrated to preserve insulin‐stimulated glucose uptake [18]. This protective effect is abolished with the addition of a CPT1 inhibitor [18], suggesting this preservation of insulin response is due to underlying effects of unAG on fatty acid oxidation. Studies from our laboratory have consistently shown that both AG and unAG stimulate fatty acid oxidation (FAO) in rat skeletal muscle [10, 18, 19, 20, 21]. Conversely, both isoforms have an acute storage promoting effect in adipose tissue, inhibiting lipolysis [9, 22, 23, 24, 25] while promoting lipogenic gene expression and intracellular lipid accumulation [7, 24] when assessed ex vivo/in vitro.

As skeletal muscle insulin resistance is strongly associated with the ectopic accumulation of lipids and subsequent intermediate formation disrupting the insulin signaling cascade [26, 27], strategies to reduce the storage of lipids may be beneficial to insulin action in this tissue. Given the acute effects of unAG on muscle fatty acid oxidation and promotion of lipid storage in adipose tissue, we hypothesized that repeated unAG administration would reduce lipid accumulation in skeletal muscle and subsequently improve insulin response. We therefore aimed to comprehensively assess the effects of short‐term repeated unAG administration on skeletal muscle and whole‐body insulin response, as well as on liver and adipose tissue substrate metabolism in lean rats. More specifically, we investigated the effects of 5 days of unAG injections on (i) basal and insulin stimulated glucose uptake and insulin signaling in oxidative and glycolytic muscle, (ii) whole body glucose management in response to insulin and pyruvate (a gluconeogenic precursor) administration, (iii) hepatic signaling pathways and content of proteins involved in hepatic glucose production, (iv) adipose tissue proteins involved in fat uptake and lipolysis, and (v) muscle and liver triacylglycerol (TAG) accumulation.

## 2 Methods

### 2.1 Materials and Reagents

Reagents, molecular weight markers, and nitrocellulose membranes for western blots were purchased from Bio‐Rad Laboratories (Mississauga, ON, Canada). Invitrogen NP40 cell lysis buffer (Cat. No. FNN0021), RestorePLUS Western Blot Stripping Buffer (Cat. No. 46430), Pierce enhanced chemiluminescence Western Blotting Substrate (Cat. No. 32106), and BCA assay reagents (Cat. No. 23227) were purchased from Thermo Fisher Scientific. Phenylmethylsulfonyl fluoride (Cat. No. P7626), protease inhibitor cocktail (Cat. No. P8340), and ponceau stain solution (Cat. No. P7170) were purchased from Millipore Sigma (Oakville, ON, Canada). Primary antibodies for phospho‐AKT Ser473 (Cat. No. 9271), phospho‐AKT Thr308 (Cat. No. 9275), AKT (Cat. No. 9272), phospho‐HSL Ser563 (Cat. No. 4139), phospho‐HSL Ser660 (Cat. No. 4126), phospho‐HSL Ser565 (Cat. No. 4137), HSL (Cat. No. 4107), alpha‐tubulin (Cat. No. 2144), CPT1B (Cat. No. 41803), and PEPCK1 (Cat. No. 12940) were purchased from Cell Signaling Technology (Danvers, MA, USA). Primary antibodies for citrate synthase (Cat. No. ab129095), cytochrome C oxidase (Cat. No. ab16056), CRF2R (Cat. No. ab104368), GLUT2 (Cat. No. ab54460), and G‐6‐Pase (Cat. No. ab319053) were purchased from Abcam (Cambridge, UK). The antibody for MCT1 was provided as a gift from Dr. Graham Holloway (University of Guelph). Goat anti‐rabbit (Cat. No. 1706515) and goat anti‐mouse (Cat. No. 1706516) secondary antibodies were purchased from BioRad Laboratories (Mississauga, ON, Canada). Lipoprotein lipase antibody (Cat. No. AF7197) and rabbit anti‐goat secondary antibody (Cat. No. HAF017) were purchased from R&D Systems (Minneapolis, MN, USA). UnAG trifluoroacetate salt (Cat. No. 4049310) was purchased from Bachem Americas (Torrence, CA, USA), D‐glucose (Cat. No. G8270), sodium bicarbonate (Cat. No. S5761), Dulbecco's Modified Eagle's Medium (Cat. No. D5030), D‐mannitol (Cat. No. 63559), bovine serum albumin (Cat. No. A7030), sodium pyruvate (Cat. No. P8574), palmitic acid (Cat. No. P0500), and 2‐deoxy‐D‐glucose (Cat. No. D8375) were purchased from Millipore Sigma. [1]‐^14^C‐Mannitol (Cat. No. ARC0127) and [1,2]‐^3^H‐2‐deoxy‐D‐glucose (ART0103) were purchased from American Radiolabeled Chemicals Inc. (St. Louis, MO, USA). EDTA (Cat. No. EDT001) and aprotinin (Cat. No. APR600) were purchased from BioShop Canada (Burlington, ON, Canada). Humulin R Insulin (Cat. No. 00586714) was purchased from Shoppers Drug Mart Inc. Non‐fat milk powder was purchased from Walmart Inc.

### 2.2 Animals and Treatment

All procedures in this study were approved by the Animal Care Committee at the University of Guelph and were in accordance with the Canadian Council on Animal Care Guidelines. For all experiments, male Sprague–Dawley rats were obtained from Charles River Canada at approximately 6 weeks of age (~150–175 g). Rats were housed in pairs and left to acclimate for 10 days, with ad libitum access to water and a standard laboratory rat diet (Teklad 2914 rodent diet, Envigo) for the duration of the study. Rats received 5 days of twice‐daily (morning, 9 a.m.; evening, 5 p.m.) subcutaneous injections of either unAG (200 𝜇g; 400 𝜇g total daily), or a vehicle control (phosphate buffered saline). This is similar to the dosage and injection protocol followed by a previous study that administered unAG [8]. Twice‐daily injections would also be potentially relevant to humans accustomed to eating two time per day. No injections were performed on the day of tissue collection to avoid any acute effects of unAG. Food intake is reported as the amount of food consumed by a cage over the period in which both animals were receiving their respective treatments (over ~56 h), divided by the number of animals in the cage (two).

### 2.3 Pyruvate Tolerance Testing

Pyruvate tolerance testing (PTT), an indirect assessment of hepatic gluconeogenic response, was conducted on the morning of the 5th treatment day, prior to that day's injections. Rats were fasted overnight. All blood glucose measurements were made using an Abbott Diabetes Care Inc. FreeStyle Libre flash glucose monitoring system glucometer and FreeStyle Precision blood glucose test strips. A small snip was made in the rat's tail, and baseline blood glucose was measured prior to pyruvate administration (*t* = 0). Rats were administered a 25% pyruvate solution at a dose of 2 g pyruvate per kg body weight, via intraperitoneal injection. Further blood glucose measurements were made at 15, 30, 45, 60, and 90 min. Area under the curve was calculated as total area above baseline.

### 2.4 Blood/Plasma Measurements

To confirm an increase in circulating ghrelin in treated animals following injections, blood samples were taken from a tail snip 30 min after the morning unAG administration on day 4 of injections. For plasma fasting ghrelin and FFA measurements, blood was collected from the tail vein just prior to surgical tissue collection. All blood samples were collected in tubes containing EDTA (1 mg/mL). Blood being used for the total ghrelin assay was additionally supplemented with aprotinin (~600–800 KIU/mL). Blood samples were left on ice for 30 min and centrifuged at 9500 *g* for 5 min to produce plasma. Plasma being prepared for the total ghrelin assay was also acidified with HCl to a final concentration of 0.05 M before being promptly frozen at −70°C. At a later date, plasma ghrelin concentrations were determined colorimetrically using a total ghrelin ELISA kit from Millipore Sigma (Cat. No. EZRGRT). Plasma FFA concentrations were determined colormetrically using an assay kit from Abcam (Cat. No. ab65341). Blood glucose was measured using a glucometer just prior to tissue collection, as well as 30 min after an IP insulin injection (1.5 U/kg) discussed later.

### 2.5 Muscle Insulin‐Stimulated Glucose Uptake Incubations

Prior to tissue collection, animals were fasted overnight. No unAG or placebo injections were given the morning of tissue collection (last injection ~5 pm on day 5). Animals were anesthetized via an intraperitoneal injection of sodium pentobarbital (6 mg/100 g BW). Soleus and extensor digitorum longus (EDL) muscle strips (~20–35 mg) were carefully removed and placed into a Dulbecco's Modified Eagles Medium (DMEM) buffer that was supplemented with sodium bicarbonate, 0.1% bovine serum albumin (BSA), 8 mM glucose, and 32 mM mannitol for 1 h to allow muscle to equilibrize. There was no insulin present in the preincubation buffer. All incubation buffers were gassed with 95% O_2_/5% CO_2_ for 20 min just prior to experiment commencement. All incubations were conducted in a 30°C shaking water bath. Following the preincubation, muscle strips were transferred to a similar wash buffer without glucose, containing 36 mM mannitol and 4 mM sodium pyruvate. Additionally, this buffer contained either no insulin or insulin at low (0.5 mU/mL) or high (10 mU/mL) concentrations. Muscles were washed in this buffer twice for 15 min. Finally, muscle was transferred into an incubation buffer for 30 min, containing 28 mM mannitol, 4 mM sodium pyruvate, and 8 mM 2‐deoxyglucose, as well as radio‐labeled [1]‐^14^C‐mannitol (~0.2 uCi/vial) and [1,2]‐^3^H‐2‐deoxy‐D‐glucose (~1 uCi per vial). Similar to the wash buffer, the incubation buffers also contained either no insulin or insulin at the low or high concentrations. Strips were then removed from the incubation buffer and rinsed briefly 2× in ice‐cold saline. Tendons were removed quickly, and the muscle was frozen in liquid nitrogen. Tendons were immediately weighed and subtracted from initial muscle weight. Muscles were removed from liquid nitrogen and solubilized in boiling 1 M NaOH for 10 min. The digest was vortexed, sampled in duplicate, added to scintillation vials containing 5 mL of Cytoscint liquid scintillation cocktail (Cat. No. 882452, MP Biomedicals, Santa Ana, CA, USA), and allowed to quench in darkness overnight. Samples were then counted 5 min per sample in a PerkinElmer Tri‐Carb LSC 4910 TR liquid scintillation counter. In addition to the above glucose uptake assay, another set of soleus muscle strips were first incubated in a high‐palmitate DMEM buffer containing 8 mM glucose, 2 mM palmitate, and 4% BSA for 4 h prior to having glucose uptake assessed at low or high insulin concentrations as described above. The only difference was that the preincubation was shortened to 15 min in an effort to reduce total incubation time. The high palmitate buffer also contained a very low dose (0.1 mU/mL) of insulin in order to prevent protein degradation that may occur during longer incubations. We were unable to perform this subset of experiments in the EDL due to tissue limitations.

### 2.6 Insulin Signaling Experiments

An additional set of soleus and EDL muscle strips were incubated in the same preincubation conditions for 1 h before being transferred to the same buffer containing no insulin, or a low (0.5 mU/mL) or a high (10 mU/mL) insulin concentration for 20 min. Muscles were then blotted dry and frozen in liquid nitrogen for further analysis by western blotting. Following surgical removal of muscles, anesthetized animals received a 1.5 U/kg insulin injection intraperitoneally. 30‐min post‐injection, blood glucose was measured to determine insulin response. Subsequently, liver, subcutaneous (SAT), and visceral (VAT) adipose tissue were removed and frozen in liquid nitrogen for further analysis of proteins in the insulin‐stimulated state via western blotting. Samples of these tissues were unable to be made prior to insulin injection in order to prevent significant bleeding during the surgery.

### 2.7 Western Blotting

Frozen tissue was chipped (20–30 mg pieces for muscle and liver, 140–160 mg pieces for adipose tissue) and homogenized using a Polytron PT 2500 E homogenizer in ice cold cell lysis buffer supplemented with a protease inhibitor and phenylmethylsulfonyl fluoride. Samples were then centrifuged at 1800 *g* for 15 min at 4°C and supernatant was removed. Supernatant from adipose tissue samples was subjected to a second round of centrifugation under the same conditions. Homogenate supernatant was removed and subjected to a bicinchoninic acid (BCA) protein quantification assay. Homogenate was then prepared with 2× laemelli sample buffer and 2‐mercaptoethanol to a final concentration of 1 mg/mL. 10 μg of protein was loaded onto 10% gels for muscle and adipose tissue blots (except 2.5 μg of protein for EDL CRF2R, 20 μg for LPL, and 12.5% gels for COXIV). Liver AKT blots were performed using 10 μg protein on 10% gels while 20 μg protein was loaded for GLUT2, PEPCK1, G‐6‐Pase, MCT1, and CRF2R blots. Gels were then subjected to electrophoresis and transferred onto nitrocellulose membranes at 100 V for 1 h and 15 min at 4°C. Membranes were blocked using 5% non‐fat skim milk (NFM) in tris‐buffered saline with Tween 20 (TBST) for 1 h at room temperature, washed for 10 min with TBST, and incubated overnight at 4°C with primary antibody (1:1000 dilution; 1:2000 for COXIV, 1:500 for GLUT2, 1:750 for PEPCK1 and G‐6‐Pase, 1:3000 for MCT1, and 1:800 for LPL). Primary antibodies were diluted in either 5% BSA or NFM in TBST depending on manufacturers' instructions. After the overnight incubation, membranes were washed with TBST for 30 min (2 × 15 min) and incubated with the appropriate secondary antibody (1:2000 in 1% NFM) for 1 h at room temperature. Membranes were washed again for 40 min (2 × 15 min TBST and 10 min 1× TBS). To image, membranes were exposed to enhanced chemiluminescence substrate for 1 min. Bands were quantified using Alpha Innotech Software. For phosphorylated proteins, membranes were stripped (~5–15 min) and reprobed with a total protein antibody, and were additionally reprobed for alpha‐tubulin to confirm equal loading. For all non‐phosphorylated proteins, ponceau stain was used for loading normalization. Protein content values for each sample were quantified relative to the average of the control sample bands (control animals, basal, if applicable) within each membrane.

### 2.8 Tissue Triacylglycerol Content Measurements

Total TAG content was measured in red and white gastrocnemius muscle, as well as in the liver using a colormetric triglyceride assay kit (Cat. No. 10010303) from Cayman Chemical Company Inc. (Ann Arbor, MI, USA), following manufacturer's instructions. Briefly, lipids were extracted from 20 to 30 mg of tissue by homogenization with a Polytron PT 2500 E homogenizer in a chloroform:isopropanol:igpal CA‐630 solution (7:11:0.1). Samples were then centrifuged for 10 min at 15 000 *g* at 4°C and supernatant was collected and dried gently under a nitrogen stream. Samples were reconstituted in the provided NP40 substitute reagent and heated to 100°C. TAGs were quantified through a glycerol colorimetric reaction at 540 nm.

### 2.9 Statistical Analysis

All data is expressed as mean ± standard deviation. Statistical significance was accepted as *p* < 0.05. All statistical analyses were conducted using GraphPad Prism 9. Initial body weight, final body weight, food intake, fasting total plasma ghrelin, fasting blood glucose, PTT AUCs, muscle CRF2R and oxidative protein content, plasma FFA levels, all liver and adipose tissue blots, and tissue TAG content were analyzed using an unpaired *t*‐test (control vs. unAG). The insulin tolerance test and blood glucose response during the PTT was analyzed using a repeated measures two‐way ANOVA (treatment × time/insulin) followed by a Bonferroni's post hoc test. Glucose uptake (low palmitate) and muscle AKT activation were analyzed using a repeated measures two‐way ANOVA (treatment × insulin concentration). Glucose uptake (high palmitate) was analyzed using an unpaired *t*‐test (control vs. unAG) within each insulin condition. All data sets were assessed for normality using the Shapiro–Wilk normality test. The Robust Regression and Outlier removal Test (set at 1% maximum discovery rate) was used to detect and remove any outliers from datasets.

## 3 Results

### 3.1 Total Plasma Ghrelin Is Increased in Circulation 30 Min Following unAG Administration

A subset of animals from both control and unAG‐treated groups had blood samples taken 30 min following unAG or vehicle control injections to confirm increased circulating total ghrelin with treatment. UnAG‐treated animals (*n* = 4) all had total plasma ghrelin concentrations that exceeded the detection limit of the assay (> 18.2 ng/mL). This represents a supraphysiological concentration. Control animals (*n* = 3) had total plasma ghrelin levels much lower, 4.9 ± 0.39 ng/mL.

### 3.2 5 Days of unAG Administration Does Not Alter Body Weight, Food Intake, or Fasting Plasma Ghrelin Levels

There was no difference in initial body weight between the control and unAG‐treated animals (*p* = 0.9913; Figure 1A). 5 days of unAG administration had no effect on final body weight (*p* = 0.9395; Figure 1B) or food intake over the experimental period (*p* = 0.6206; Figure 1C). To confirm that short‐term chronic unAG administration did not impact endogenous ghrelin levels, we measured fasting plasma total ghrelin levels at the time of tissue collection (~16 h since last injection). There was no difference in fasting plasma total ghrelin levels between control (*n* = 10; 2.684 ± 1.011 ng/mL) and unAG‐treated animals (*n* = 8; 2.904 ± 0.7528 ng/mL; *p* = 0.6167).

**FIGURE 1:**
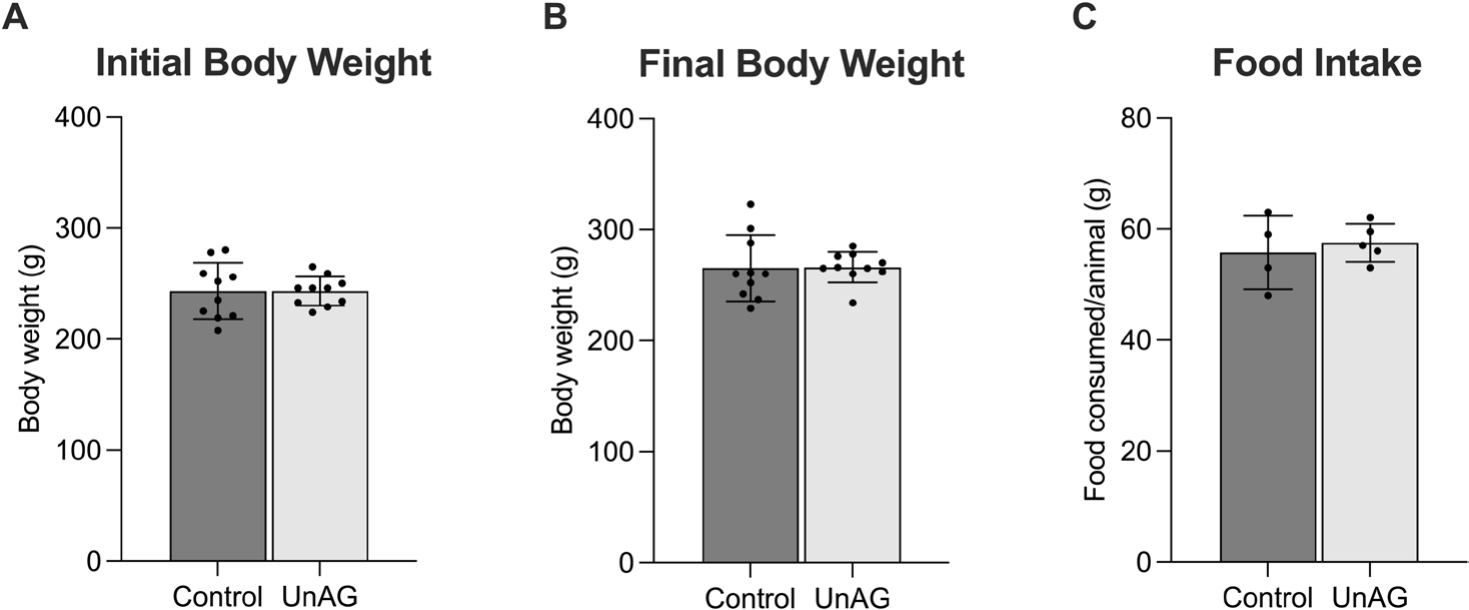
Body weight and food intake. Initial body weight of animals prior to study commencement (A), final body weight after 5 days of twice‐daily subcutaneous injections of 200 μg of unAG or a vehicle control (B), and estimated food consumed per animal, calculated as food consumed per cage over the course of the injection period when both animals in a cage were receiving treatment (~56 h), divided by the number of animals in the cage (two; C). Data are expressed as mean ± standard deviation. Initial and final body weight (*n* = 10/group) and food intake (*n* = 4–5/group) were analyzed using unpaired *t*‐tests. *p* < 0.05 was considered statistically significant.

### 3.3 UnAG Administration Reduces Gluconeogenic Response to Pyruvate Administration

Fasting blood glucose did not differ between treatment groups (*p* = 0.9650; Figure 2A). Insulin significantly lowered blood glucose (*p* < 0.0001), but this did not differ between treatment groups (*p* = 0.2131; Figure 2B). There was a significant interaction effect between unAG treatment and time on blood glucose over the 90‐min PTT (*p* = 0.0434; Figure 2C). Glucose area under the curve values were also significantly lower in unAG‐treated animals (*p* = 0.0299; Figure 2D).

**FIGURE 2:**
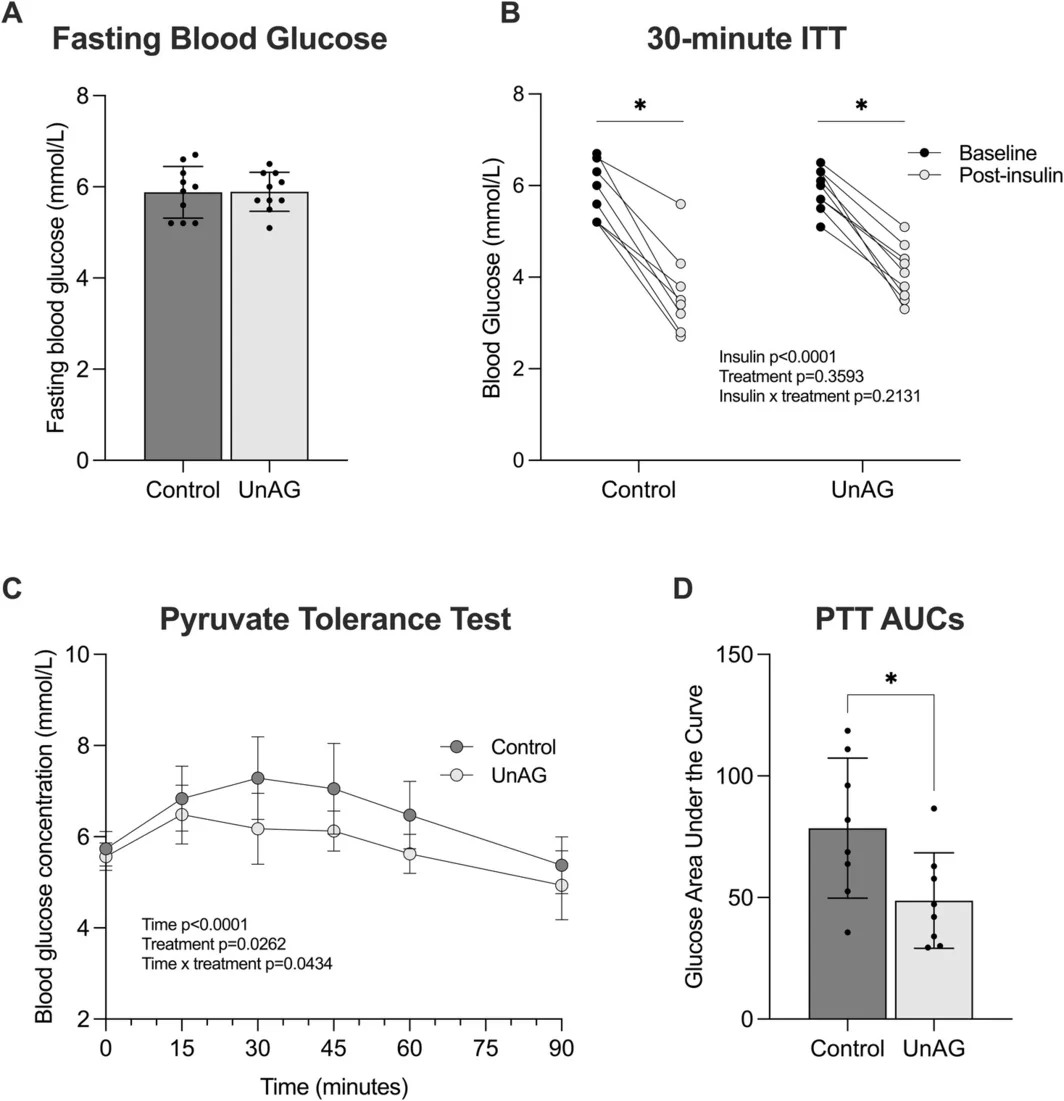
Whole‐body glucose homeostasis and pyruvate tolerance. Fasting blood glucose (A), 30‐min insulin tolerance test (ITT; 1.5 U/kg; B), blood glucose over a pyruvate tolerance test (PTT; C), and PTT blood glucose area under the curve (AUC; D) following 5 days of twice‐daily subcutaneous injections of 200 μg of unAG or a vehicle control. Data are expressed as mean ± standard deviation. Fasting blood glucose (*n* = 10/group) and PTT AUCs (*n* = 8/group) were analyzed using an unpaired *t*‐test. Insulin tolerance test (*n* = 8–10/group) and blood glucose over the PTT (*n* = 8/group) were analyzed using a repeated measures two‐way ANOVA (treatment × insulin and treatment × time, respectively), followed by a Bonferroni post hoc test if a significant interaction effect was found. *p* < 0.05 was considered statistically significant. *Indicates significance.

### 3.4 Insulin‐Stimulated Glucose Uptake and AKT Activation in Oxidative and Glycolytic Muscle Is Not Altered After 5 Days of unAG Administration

Glucose uptake did not differ between treatment groups when measured under basal, low‐insulin (0.5 mU/mL), or high‐insulin (10 mU/mL) conditions, in soleus (*p* = 0.3717; Figure 3A) or EDL (*p* = 0.4947; Figure 3B) muscle. There was a significant overall effect of insulin to increase glucose uptake in both muscle types (*p* < 0.0001; Figure 3A,B). AKT activation at both pAKT^Ser473^ (soleus *p* = 0.4394; EDL *p* = 0.5834) and pAKT^Thr308^ (soleus *p* = 0.2347; EDL *p* = 0.3255) did not differ between treatment groups when measured in response to basal, low‐insulin (0.5 mU/mL), or high‐insulin (10 mU/mL) conditions, in soleus (Figure 3C) or EDL (Figure 3E) muscle. There was a significant overall effect of insulin to increase AKT activation at both phosphorylation sites in both the soleus and EDL (*p* < 0.0001; Figure 3C,E).

**FIGURE 3:**
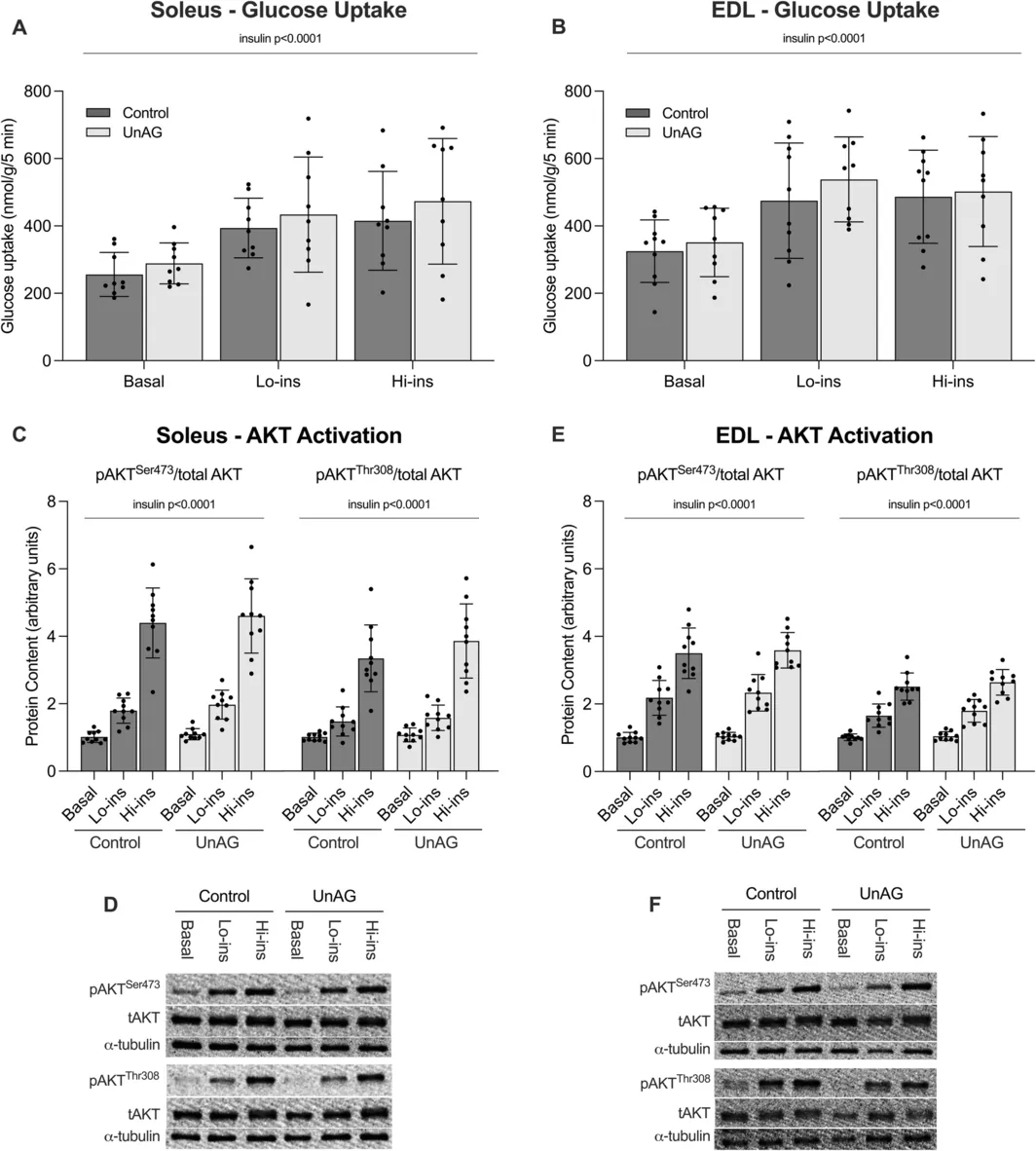
Insulin‐stimulated glucose uptake and AKT activation in oxidative and glycolytic muscle. Glucose uptake and AKT activation at Ser473 and Thr308 in soleus (A and C, respectively) and EDL (B and E, respectively) muscle in response to basal, low (Lo‐ins, 0.5 mU/mL)‐, or high (Hi‐ins, 10 mU/mL)‐insulin conditions following 5 days of twice‐daily subcutaneous injections of 200 μg of unAG or a vehicle control. Representative blots are shown (D and F). Glucose uptake (*n* = 9–10/group) and AKT activation (*n* = 10/group) are expressed as mean ± standard deviation and analyzed using a repeated measures two‐way ANOVA (treatment × time). *p* < 0.05 was considered statistically significant.

### 3.5 5 Days of unAG Administration Does Not Protect Against Acute High‐Fat Induced Impairments to Insulin‐Stimulated Glucose Uptake in Soleus Muscle

A 4‐h pre‐exposure to a high‐palmitate (2 mM) environment resulted in lowered insulin‐stimulated glucose uptake under both low (0.5 mU/mL) or high (10 mU/mL) insulin conditions in soleus muscle (Figure 4). 5 days of unAG treatment did not improve glucose uptake response to low (*p* = 0.1747) or high (*p* = 0.9348) concentrations of insulin (Figure 4).

**FIGURE 4:**
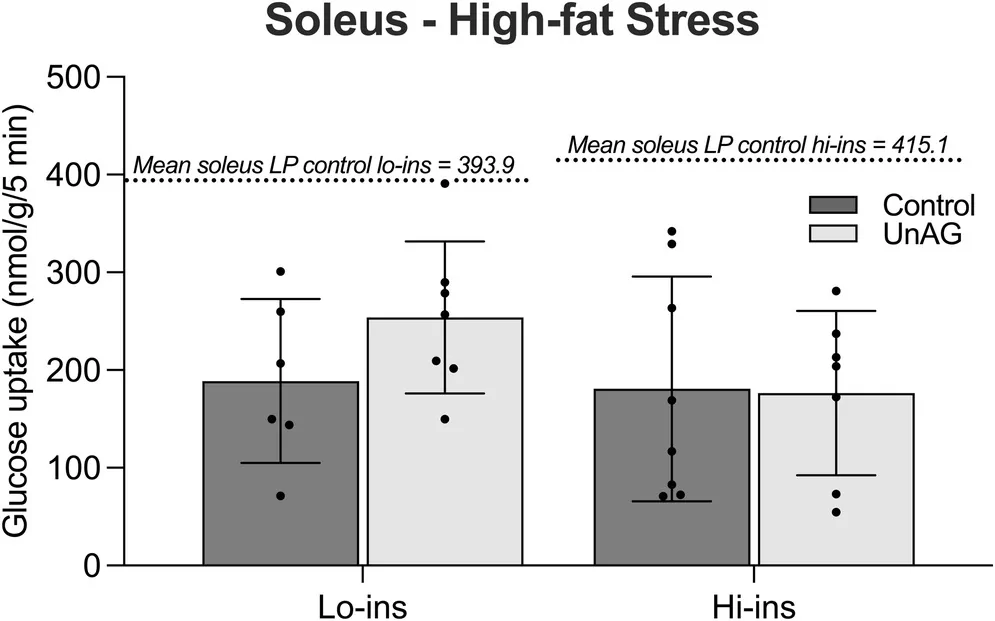
Glucose uptake following high‐fat stress. Glucose uptake measurements following a 4‐h pre‐exposure to a high‐palmitate (2 mM) buffer under low (Lo‐ins; 0.5 mU/mL) or high (Hi‐ins; 10 mU/mL)‐insulin conditions, following 5 days of twice‐daily subcutaneous injections of 200 μg of unAG or a vehicle control. The dotted lines represent mean glucose uptake levels in response to the respective insulin condition under low‐palmitate conditions, as measured in Figure 4. Data (*n* = 6–8/group) are expressed as mean ± standard deviation and were analyzed using unpaired *t*‐tests within each insulin condition. *p* < 0.05 was considered statistically significant.

### 3.6 5 Days of unAG Administration Does Not Alter Protein Content of Mitochondrial Markers in Oxidative or Glycolytic Muscle

Treatment with unAG did not alter CS (soleus *p* = 0.6235; EDL *p* = 0.3516) or CPT1B (soleus *p* = 0.2175; EDL *p* = 0.5735) protein content in soleus or EDL muscle (Figure 5A,C). COXIV protein content did not differ between groups in the soleus (*p* = 0.1742; Figure 5A), however, it was significantly reduced in the EDL of unAG‐treated animals (*p* = 0.0188; Figure 5C).

**FIGURE 5:**
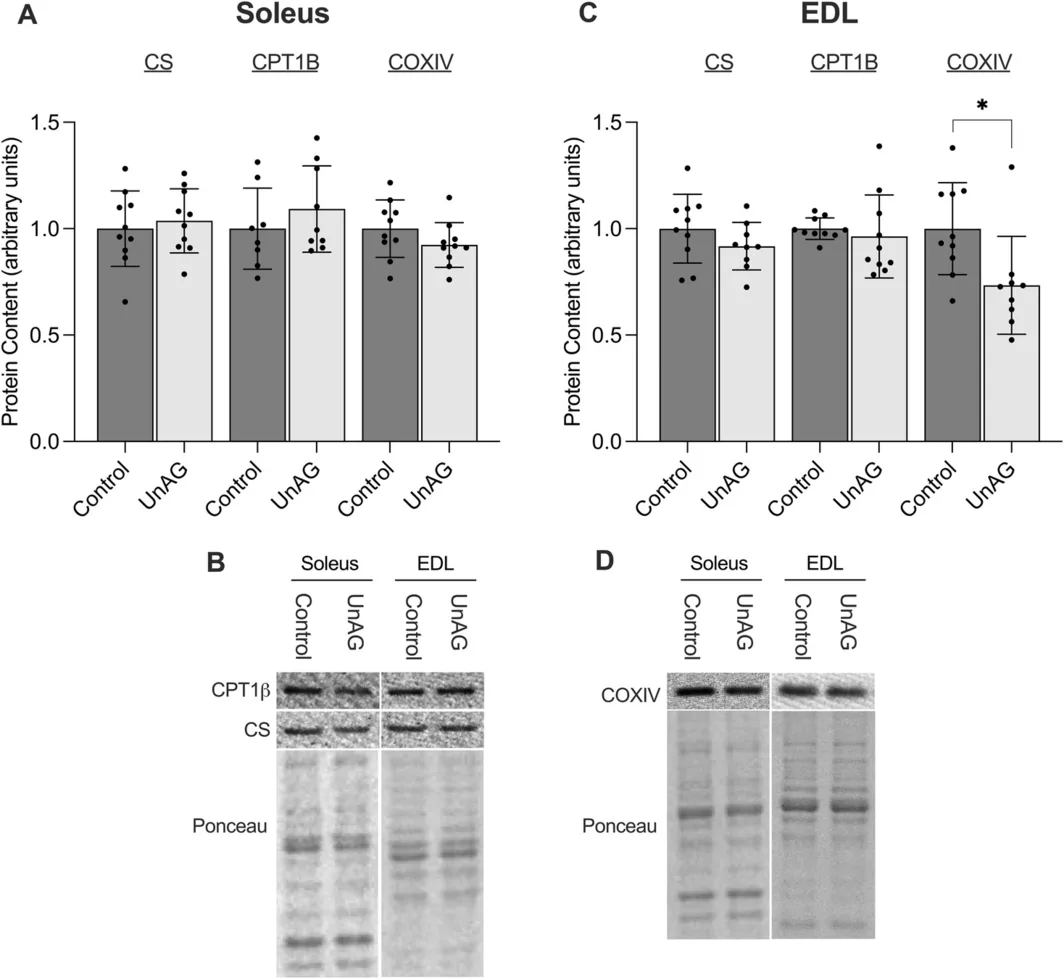
Muscle mitochondrial marker protein content. Citrate synthase (CS), carnitine palmitoyltransferase 1B (CPT1B), and cytochrome c oxidase subunit 4 (COXIV) protein content in soleus (A) and EDL (C) muscle following 5 days of twice‐daily subcutaneous injections of 200 μg of unAG or a vehicle control. Data (*n* = 8–10/group) is expressed as mean ± standard deviation, and representative blots are shown (B and D). Data is analyzed using unpaired *t*‐tests. *p* < 0.05 was considered statistically significant. *Indicates significance.

### 3.7 5 Days of unAG Administration Does Not Alter Hepatic AKT Activation in Response to an Insulin Bolus or Content of Hepatic Glucose Production‐Associated Proteins

AKT activation at both pAKT^Ser473^ (*p* = 0.3958) or pAKT^Thr308^ (*p* = 0.7698) was similar between treatment groups in response to an intraperitoneally administered insulin bolus (1.5 U/kg; Figure 6A). Additionally, protein content of MCT1 (*p* = 0.4306), PEPCK (*p* = 0.5728), G‐6‐Pase (*p* = 0.9601), and GLUT2 (*p* = 0.6304) did not change with unAG treatment (Figure 6C).

**FIGURE 6:**
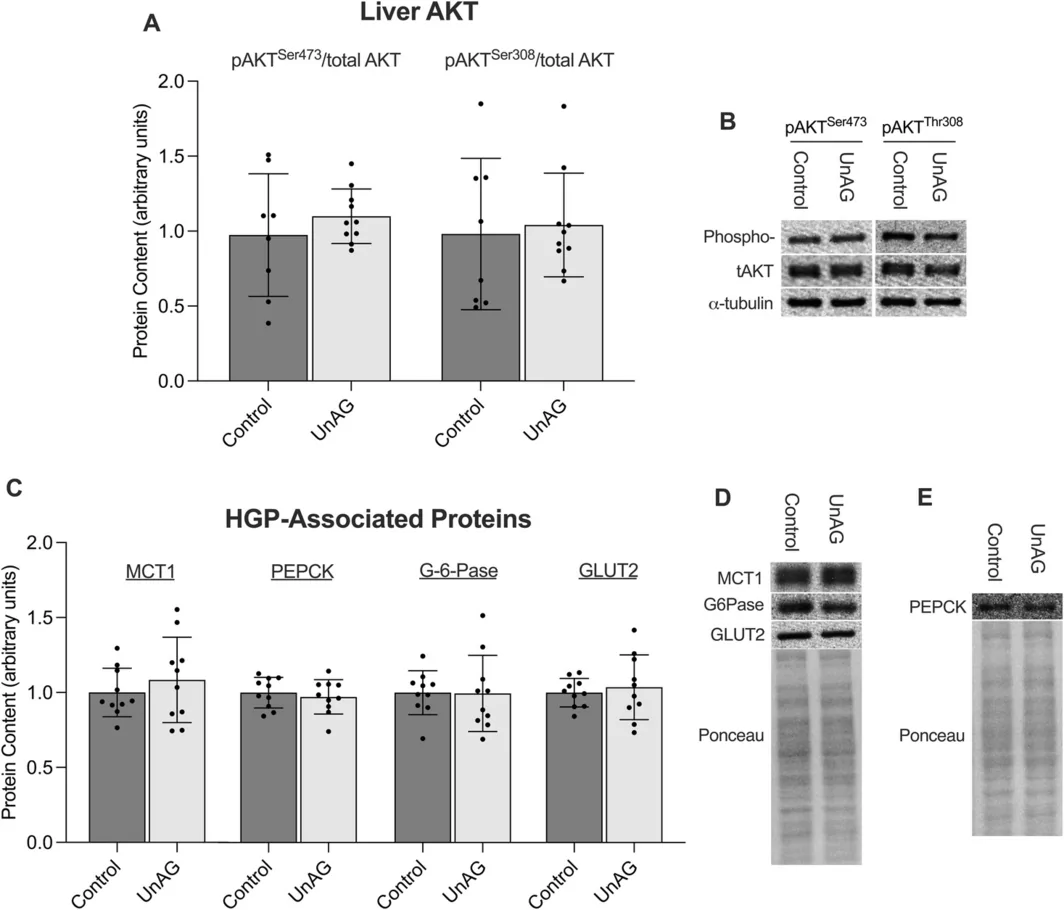
Liver AKT signaling and HGP‐associated protein content. Hepatic AKT activation at Ser473 and Thr308 30 min after the administration of an intraperitoneal bolus of insulin (1.5 U/kg; A), as well as content of hepatic glucose production (HGP)‐associated proteins (Monocarboxylate Transporter 1 [MCT1], phosphoenolpyruvate carboxykinase [PEPCK], glucose‐6‐phosphatase [G‐6‐Pase], and glucose transporter 2 [GLUT2]; C) following 5 days of twice‐daily subcutaneous injections of 200 μg of unAG or a vehicle control. AKT activation (*n* = 8–10/group) and HGP‐associated protein content (*n* = 10/group) are expressed as mean ± standard deviation, and representative blots are shown (B, D, and E). Data is analyzed using unpaired *t*‐tests. *p* < 0.05 was considered statistically significant.

### 3.8 Subcutaneous and Visceral Adipose Tissue AKT and HSL Phosphorylation Are Unaffected, but LPL Content Tends to Be Increased by 5 Days of unAG Injections

UnAG treatment did not alter lipoprotein lipase (LPL) content in SAT (*p* = 0.9304), but tended to increase it in VAT (*p* = 0.0736; Figure 7A). UnAG treatment did not alter AKT phosphorylation at either Ser473 (SAT *p* = 0.5062; VAT *p* = 0.6114) or Thr308 (SAT *p* = 0.9828; VAT *p* = 0.9706) sites in response to an intraperitoneally administered insulin bolus (1.5 U/kg; Figure 7C) in either SAT or VAT. Additionally, HSL phosphorylation at the two stimulatory (HSL^Ser563^ [SAT *p* = 0.7697; VAT *p* = 0.5397] or pHSL^Ser660^ [SAT *p* = 0.7397; VAT *p* = 0.8736]) and inhibitory (pHSL^Ser565^ [SAT *p* = 0.2579; VAT *p* = 0.2295]) sites (Figure 7E), as well as fasting free fatty acid levels (*p* = 0.3708; Figure 7G), did not differ between treatment groups.

**FIGURE 7:**
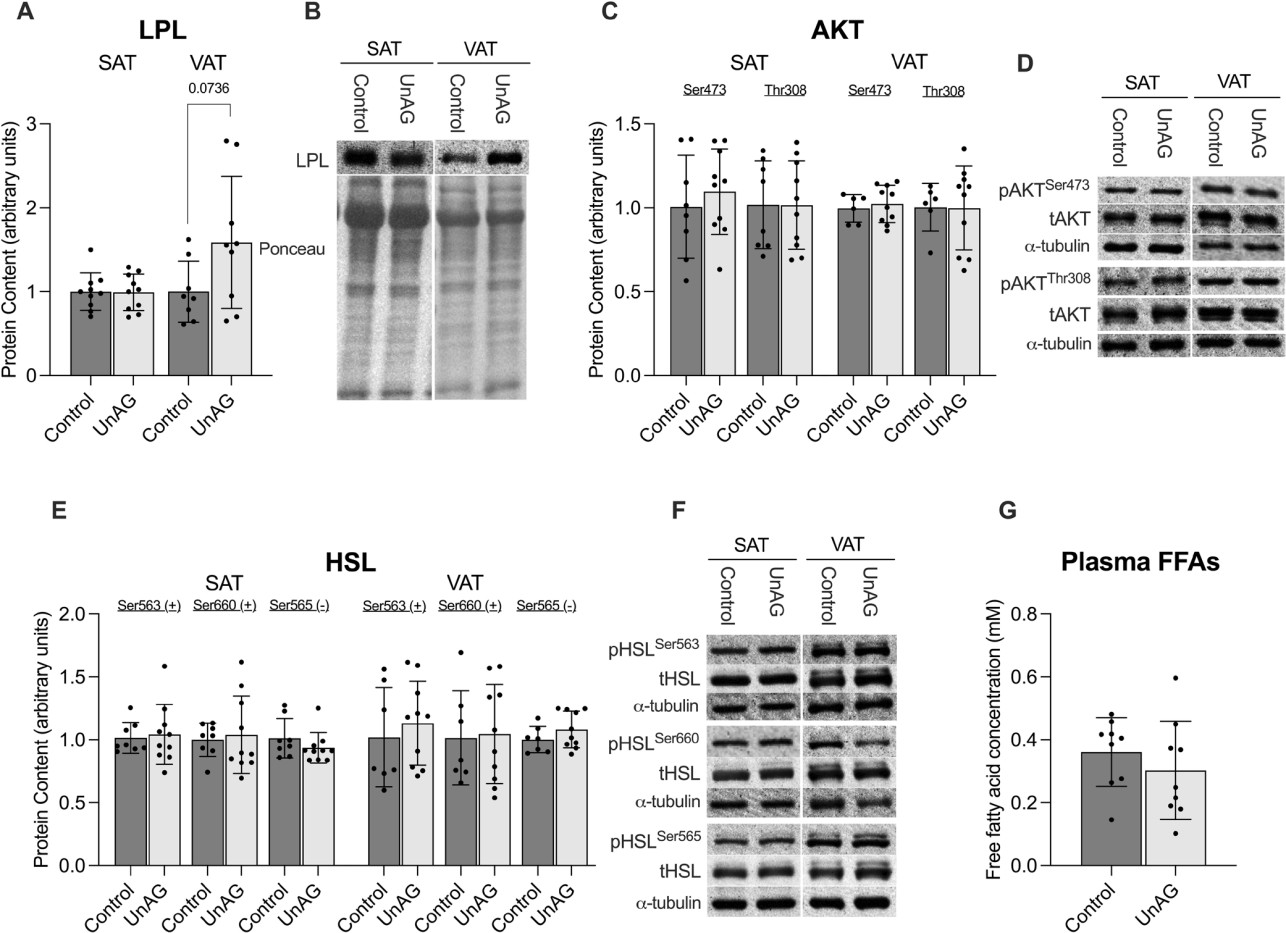
Adipose tissue LPL content, AKT and HSL signaling, and circulating FFAs. Subcutaneous (SAT) and visceral (VAT) adipose tissue lipoprotein lipase (LPL) content (A), AKT activation at Ser473 and Thr308 (C) and HSL phosphorylation at Ser563, Ser660, and Ser565 (E) 30 min after the administration of an intraperitoneal bolus of insulin (1.5 U/kg), and fasting plasma circulating free fatty acid (FFAs) concentration (G) following 5 days of twice‐daily subcutaneous injections of 200 μg of unAG or a vehicle control. Data is expressed as mean ± standard deviation, and representative blots are shown (B, D, and F). LPL protein content (*n* = 8–10), AKT activation (*n* = 6–10), HSL phosphorylation (*n* = 7–10), and plasma FFA concentrations (*n* = 9) were all analyzed using unpaired *t*‐tests. *p* < 0.05 was considered statistically significant.

### 3.9 5 Days of unAG Administration Does Not Alter Triacylglycerol Content in Oxidative or Glycolytic Muscle, or in the Liver

UnAG treatment did not alter TAG content in white (*p* = 0.8463) or red (*p* = 0.9212) gastrocnemius muscle, or in the liver (*p* = 0.2317; Figure 8).

**FIGURE 8:**
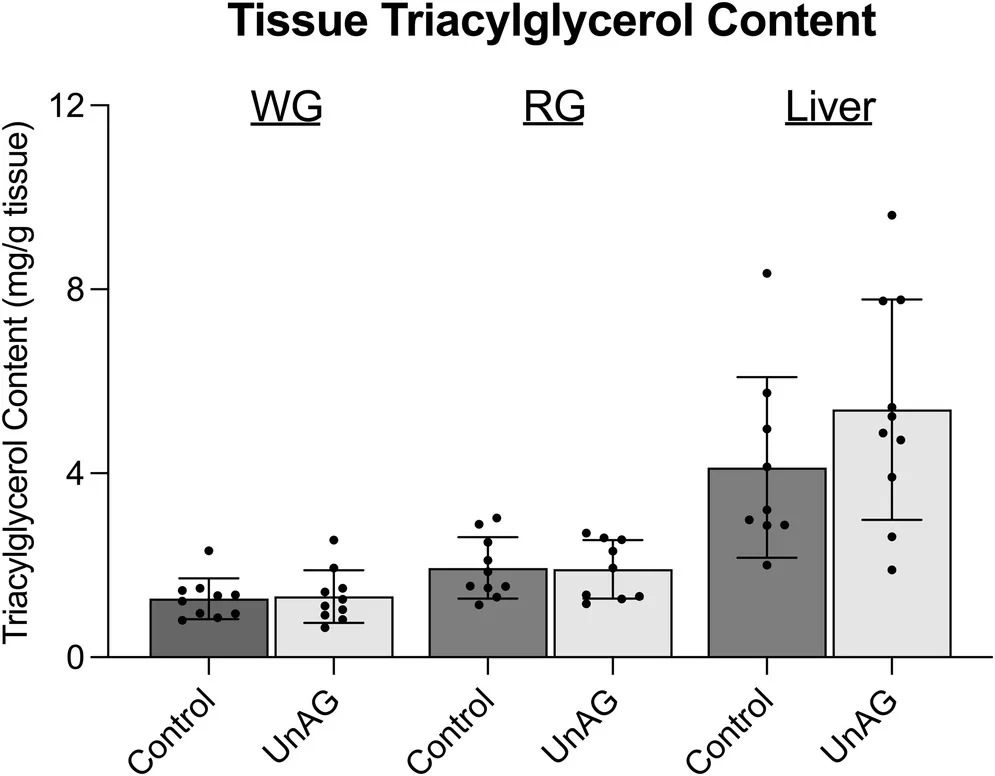
Tissue TAG content. Tissue triacylglycerol (TAG) content in white (WG) and red (RG) gastrocnemius muscle, and in the liver, following 5 days of twice‐daily subcutaneous injections of 200 μg of unAG or a vehicle control. Data (*n* = 9–10/group) is expressed as mean ± standard deviation and was analyzed using unpaired *t*‐tests within a tissue type. *p* < 0.05 was considered statistically significant.

### 3.10 5 Days of unAG Administration Does Not Alter Protein Content of the Putative unAG Receptor, Corticotropin‐Releasing Factor Receptor 2, in Skeletal Muscle or the Liver

We measured the putative skeletal muscle unAG receptor, corticotropin‐releasing factor receptor 2 (CRF2R) [28, 29], in skeletal muscle as well as in other tissues in order to determine whether repeated exogenous unAG treatment might cause its downregulation. An initial western blot was performed (*n* = 1, one control and one unAG‐treated; 10 μg loaded; Figure 9A) on each of the soleus, EDL, liver, SAT, and VAT tissue samples to determine relative content of CRF2R in each tissue type and to optimize blotting conditions for tissues with sufficient expression. Based on this, we proceeded with CRF2R measurements in the soleus (10 μg loaded), EDL (2.5 μg loaded), and liver (20 μg loaded), while both adipose tissue depots were excluded due to the very low expression levels and unreliable detectability. There was no effect of 5 days of unAG content in the soleus (*p* = 0.5907), EDL (*p* = 0.4547), or the liver (*p* = 0.8304; Figure 9C).

**FIGURE 9:**
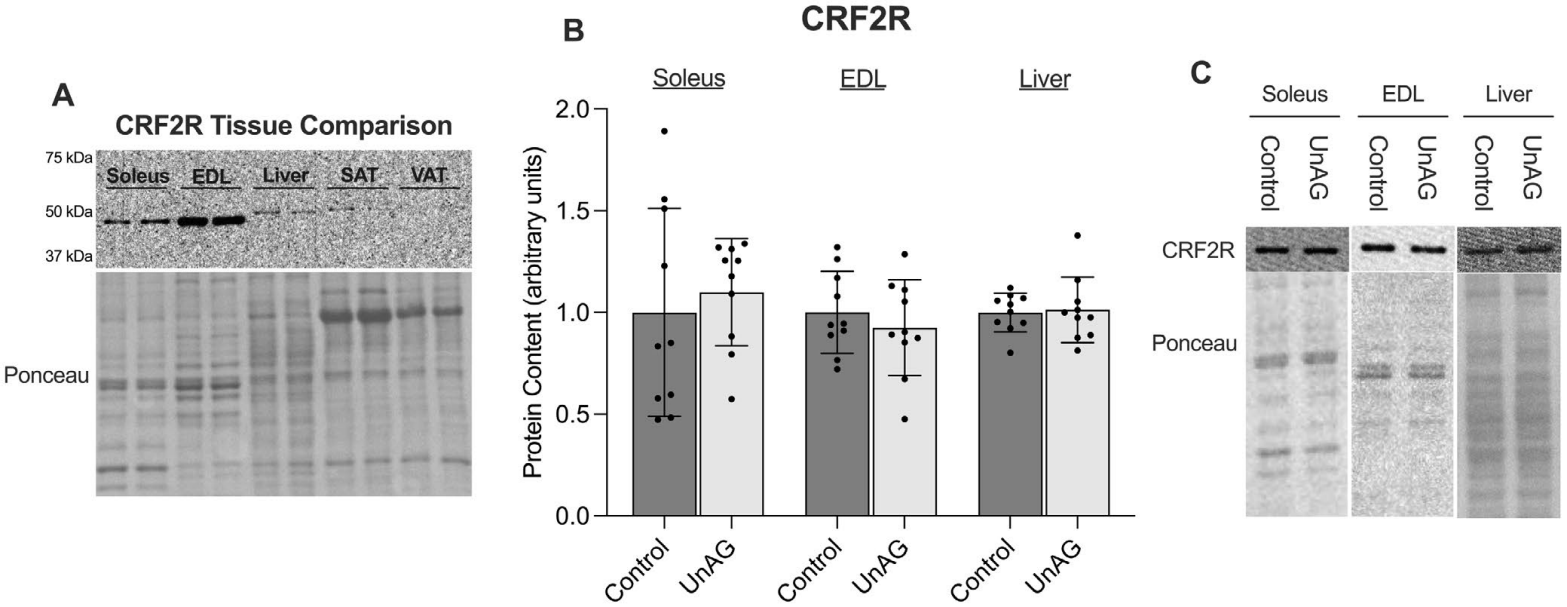
CRF2R protein content. An initial tissue comparison western blot of CRF2R protein content in the soleus, extensor digitorum longus (EDL), liver, subcutaneous adipose tissue (SAT), and visceral adipose tissue (VAT; A), and CRF2R protein content in the soleus, EDL, and liver (B) following 5 days of twice‐daily subcutaneous injections of 200 μg of unAG or a vehicle control. Representative blots are shown (C). Data (*n* = 10) are expressed as mean ± standard deviation and were analyzed using unpaired *t*‐tests. *p* < 0.05 was considered statistically significant.

## 4 Discussion

### 4.1 Overview

The present study aimed to assess whether 5 days of twice‐daily unAG administration would improve skeletal muscle insulin response, and whether this would be associated with a reduction in muscle lipid accumulation. We also broadened our analyses by assessing whole body response to pyruvate and insulin injections, as well as key proteins in liver and adipose tissue involved in gluconeogenesis and lipolysis, respectively. Contrary to our hypothesis, repeated unAG administration did not improve insulin response or reduce lipid content in oxidative or glycolytic muscle. However, UnAG treatment improved the glycemic response to a pyruvate bolus, potentially indicating a reduced gluconeogenic response; this was not associated with changes in hepatic AKT response to insulin, content of glucose production‐related proteins (MCT‐1, PEPCK, G‐6‐Pase, GLUT2), or liver lipid content. In adipose tissue, AKT response to insulin, as well as HSL phosphorylation, and LPL protein content remained unchanged with unAG. Overall, these findings indicate that repeated short‐term unAG administration improves pyruvate tolerance but does not induce other chronic alterations in insulin responsive peripheral tissues in lean rats.

### 4.2 Alterations in Skeletal Muscle Insulin Response and Lipid Metabolism With unAG

UnAG has been demonstrated by our laboratory to acutely stimulate FAO ex vivo in isolated oxidative and glycolytic rat skeletal muscle [10, 18, 19, 20, 21]. UnAG also exhibits antilipolytic effects [9, 10, 22, 23] and promotes lipogenesis [7] in adipose tissue. Given this, we hypothesized that repeated unAG administration would be beneficial for skeletal muscle insulin response by diverting circulating lipids towards adipose tissue while simultaneously increasing FAO in muscle, resulting in reduced ectopic lipid accumulation and improved insulin signaling.

One previous study using a similar unAG administration protocol (4 days of twice‐daily 200 μg unAG injections) demonstrated improved glucose uptake in response to a relatively low insulin dose (600 pmol/L, or equivalent ~0.086 mU/mL), as well as increased basal phosphorylation of insulin signaling proteins in rats [8]. These findings contrast with the current work which found no change in skeletal muscle insulin response basally, or under low (0.5 mU/mL) or high (10 mU/mL) insulin concentrations. One potentially confounding factor in the previous study is that metabolic assessments were made 3 h after the final unAG injection, meaning that an acute unAG effect cannot be ruled out. In the current study, metabolic assessments were performed ~16‐h after the final injection. Importantly, our lab has previously identified that unAG's ability to stimulate FAO in skeletal muscle is sustained for at least 2–3 h after hormonal exposure [20], supporting the idea that unAG may still be exerting acute effects 3 h after administration. It is also possible that technical differences such as the method of determining muscle glucose uptake may have affected the results. In the previous study [8], glucose uptake was determined using non‐radioactive 2‐deoxyglucose, whereas in the current study radiolabeled tritiated 2‐deoxyglucose was used.

### 4.3 UnAG and Hepatic Glucose Metabolism

A novel finding of the present work is that 5 days of unAG treatment improves pyruvate tolerance, observed by a reduced glycemic response to a PTT in unAG‐treated animals. A reduction in glycemic response to a PTT can serve as an indirect marker of reduced hepatic gluconeogenesis. This reduction in glycemic response to pyruvate is in line with previous work demonstrating unAG treatment to acutely reduce glucose output from isolated primary hepatocytes [6]. In contrast, AG has a stimulatory effect on hepatic glucose production [6, 30, 31], likely mediated by a reduction in AKT activation and subsequent upregulation of PEPCK and G6Pase [30, 31]. This is also observed chronically, with 4 days of AG administration reducing hepatic AKT activation [15] and increasing G6Pase expression [14]. UnAG antagonizes the stimulatory effect of AG on EGP in primary hepatocytes [6], which directed our investigation towards the insulin‐mediated transcriptional regulation of gluconeogenesis. We therefore explored the underlying mechanism of improved pyruvate tolerance by assessing (i) hepatic AKT response to an insulin bolus, and (ii) the content of proteins involved in hepatic glucose production. We found no difference in AKT activation in response to an insulin bolus between unAG and control treated animals. It should be acknowledged that a high insulin dose was administered; it is possible that we may have observed differences in insulin signaling with a smaller insulin concentration. Additionally, the protein content of MCT1 (transports pyruvate into hepatocytes), PEPCK and G6Pase (key steps of gluconeogenesis), and GLUT2 (exports newly formed glucose from hepatocytes into circulation) were unaltered by repeated unAG treatment.

The unchanged content of HGP‐associated proteins does not necessarily preclude a change in gluconeogenic flux. Gluconeogenesis is largely regulated at the transcriptional level, particularly PEPCK and G6Pase [32]. However, other levels of regulation exist beyond protein translation, such as allosteric regulation of other key gluconeogenic enzymes like pyruvate carboxylase and fructose‐1‐6‐biphosphatase. It is also possible that unAG is reducing the glycemic response to pyruvate by altering its partitioning towards other fates within the liver, such as oxidation or glycogen synthesis, which to our knowledge remains unassessed in response to unAG. Finally, increased glucose clearance in other tissues could contribute to a reduced glycemic response to a pyruvate bolus, although our own results indicate this is not likely to be skeletal muscle. Future work should directly measure endogenous glucose output from the liver following short‐term unAG administration to determine whether the reduced glycemic response to pyruvate is in fact the result of chronic changes to hepatic gluconeogenesis.

### 4.4 Regulation of Adipose Tissue Metabolism by unAG

Both ghrelin isoforms have been shown to acutely inhibit lipolysis in adipose tissue under stimulatory conditions [9, 10, 22, 23], likely through increased PDE‐mediated degradation of cAMP [23] and subsequently reduced HSL phosphorylation [9, 10]. In addition, unAG increases AKT activation in preadipocytes, and inhibition of insulin signaling proteins or PDE3B prevents unAG's suppressive effects on isoproterenol‐stimulated lipolysis [23]. Together, this indicates that unAG may inhibit lipolysis through increased insulin signaling. Therefore, we examined whether repeated unAG administration elicits chronic changes to the response of adipose tissue to insulin. We assessed AKT activation at its two main regulatory sites (Ser473 and Thr308) and HSL phosphorylation at two stimulatory sites known to be downstream of PKA/cAMP signaling (Ser563 and 660) [33, 34] as well as its negative regulatory site (Ser565) [35]. We found that short‐term unAG treatment did not alter AKT or HSL phosphorylation in response to an insulin bolus. Additionally, we measured fasting plasma fatty acids as an approximate index of lipolysis and did not find any differences between groups. These findings suggest that repeated unAG administration does not alter adipose tissue lipolytic response to insulin. However, it is important to note that unAG's suppressive effects on lipolysis have only been demonstrated under stimulatory conditions [9, 10, 22, 23], with unAG having no effect on basal lipolysis [9, 10, 22]. Future studies should examine the impact of chronic unAG administration on lipolytic capacity under stimulatory conditions (isoproterenol, epinephrine). This was not feasible in the present study.

Both ghrelin isoforms have also been found to have lipogenic effects on adipose tissue. Although the effects of AG on lipid storage have been more thoroughly characterized [7, 36, 37, 38], one study by Rodríguez et al. demonstrated that unAG increased the mRNA expression of the lipogenic transcription factors PPARy (*PPARG*) and SREBP1 (*SREBF1*), increased content of lipid storage proteins (ACC, LPL, FAS, and perilipin), and increased intracellular TAG content in human visceral adipocytes [7]. While assessing chronic changes in lipogenic pathways with unAG was not a primary endpoint of the present work, we measured total LPL protein content in adipose tissue following treatment. We found a trend (*p* = 0.0736) towards increased LPL protein content in VAT. LPL and its regulators have long been identified as key modulators of lipid partitioning depending on nutritional state, specifically with increased adipose LPL activity postprandially directing TAGs away from muscle to adipose tissue for storage [39, 40, 41]. Interestingly, adipose tissue‐specific LPL overexpression has been found to result in improved whole‐body glucose tolerance and insulin response [42], and genetic ablation of adipose tissue ANGPTL4, which inhibits LPL, reduces TAG and DAG content in both liver and muscle tissue while also improving glucose tolerance [43]. Importantly, both these genetic models found these beneficial effects in animals challenged with a high‐fat diet [42, 43] suggesting that future studies should examine the effects of unAG administration on LPL content in animals under metabolic stress.

### 4.5 Physiological Role of unAG in Postprandial Lipid Handling and Protection From High‐Fat Feeding

We have recently proposed that unAG may act as a preprandial signal to prepare peripheral tissues for incoming meal‐derived lipids [44], inhibiting lipolysis [9, 10, 22, 23] and activating storage pathways [7] in adipose tissue, while stimulating oxidation in muscle [10, 18, 19, 20, 21]. A recent study from our lab demonstrated the stimulatory effects of unAG on skeletal muscle FAO to persist for 2–3 h after tissue is removed from direct hormonal exposure [20]. When we align this with meal‐derived lipids typically peaking in circulation 2–3 h following a meal [45], it is tempting to suggest that a preprandial rise in unAG may help to facilitate disposal of lipids in the postprandial period. However, it remains to be assessed whether unAG's acute lipogenic effects on adipose tissue are similarly sustained. Given this suggested physiological role, a potential effect of unAG on lipid partitioning may be highly relevant in the immediate postprandial state when circulating meal‐derived lipids are elevated.

We utilized a lean, healthy rat model for these initial experiments. This model was selected because (i) previous work demonstrated a similar protocol would be sufficient to see an effect with respect to substrate metabolism in lean, healthy animals [8, 14, 15]; and (ii) to establish a baseline for unAG's effects for future investigation using a metabolically‐challenged, HFD animal model. High‐fat feeding is known to result in several hallmark metabolic disturbances to variables assessed in the present work, such as reduced skeletal muscle insulin response [46, 47, 48], impaired suppression of endogenous glucose output [47, 49, 50, 51], elevated circulating lipids [48, 52], and increased hepatic and intramuscular lipid storage [48, 50, 51]. It is possible that in high‐fat fed animals, the combination of metabolic dysfunction, increased dietary lipid intake, and reduced postprandial metabolic flexibility [53] may make any potential effects of unAG on lipid partitioning more apparent. In support of this, we have previously found that despite having no direct effects on insulin response in skeletal muscle [17], unAG is able to preserve insulin‐stimulated glucose uptake in muscle incubated in a high‐palmitate buffer, which typically induces impairments to the action of insulin [18]. The present study did not find repeated unAG administration to induce chronic changes in skeletal muscle that protect against high‐palmitate induced impairments to insulin‐stimulated glucose uptake; however, protective effects of unAG may require a multi‐tissue in vivo action or a chronic exposure to a high‐fat environment. In line with this, transgenic overexpression of unAG improves whole‐body insulin response in mice fed a high‐fat diet, and this improvement was greater than what was observed in chow‐fed animals [8].

## 5 Limitations

The present study is limited in its male‐only design. Male rats were used for continuity with our own previous studies as well as others. However, given the known sex differences in substrate metabolism, confirming our current findings in female rats is important. In addition, all liver and adipose tissue AKT and HSL phosphorylation data represent only the insulin‐stimulated state as tissues could not be sampled prior to insulin injections due to bleeding concerns. Finally, the results of our pyruvate tolerance test do not conclusively demonstrate altered hepatic gluconeogenesis. Such experimental work would require further studies utilizing radiolabelled precursors and are beyond the scope of the present study.

## 6 Conclusion

Overall, the present study demonstrates that short‐term repeated unAG administration improves pyruvate tolerance in lean healthy rats. Contrary to our hypothesis, this was not associated with any changes in skeletal muscle insulin response, TAG accumulation in the liver or muscle, or hepatic and adipose tissue insulin signaling. In conclusion, unAG does not seem to induce chronic metabolic changes in a metabolically healthy phenotype, despite its acute effects on peripheral tissue metabolism. Future work should aim to determine whether under metabolically stressed conditions (i.e., chronic high‐fat feeding), repeated unAG treatment would improve skeletal muscle insulin response via its multi‐tissue effects observed acutely.

## Author Contributions

Nicole M. Notaro: conceptualization, methodology, investigation, writing – original draft preparation, writing – review and editing. Sofia F. Prizio: Investigation, writing – review and editing. Brielle R. Caruso: investigation, writing – review and editing. David J. Dyck: conceptualization, methodology, investigation, writing – original draft preparation, writing – review and editing, supervision, funding acquisition.

## Funding

This work was supported by the Natural Sciences and Engineering Research Council of Canada (NSERC) (2022‐03425).

## Conflicts of Interest

The authors declare no conflicts of interest.

## Data Availability

All the data that supports the findings of this study are available in the results of this article.
